# The *Agropyron cristatum* karyotype, chromosome structure and cross-genome homoeology as revealed by fluorescence in situ hybridization with tandem repeats and wheat single-gene probes

**DOI:** 10.1007/s00122-018-3148-9

**Published:** 2018-08-01

**Authors:** Mahmoud Said, Eva Hřibová, Tatiana V. Danilova, Miroslava Karafiátová, Jana Čížková, Bernd Friebe, Jaroslav Doležel, Bikram S. Gill, Jan Vrána

**Affiliations:** 1 Institute of Experimental Botany, Center of the Region Haná for Biotechnological and Agricultural Research, Šlechtitelů 31, 78371 Olomouc, Czech Republic; 2 Field Crops Research Institute, Agricultural Research Centre, 9 Gamma Street, Giza, Cairo 12619 Egypt; 3 Wheat Genetics Resource Center, Kansas State University, 1712 Claflin Road, 4024 Throckmorton PSC, Manhattan, KS 66506 USA

## Abstract

**Key message:**

Fluorescence in situ hybridization with probes for 45 cDNAs and five tandem repeats revealed homoeologous relationships of *Agropyron cristatum* with wheat. The results will contribute to alien gene introgression in wheat improvement.

**Abstract:**

Crested wheatgrass (*Agropyron cristatum* L. Gaertn.) is a wild relative of wheat and a promising source of novel genes for wheat improvement. To date, identification of *A. cristatum* chromosomes has not been possible, and its molecular karyotype has not been available. Furthermore, homoeologous relationship between the genomes of *A. cristatum* and wheat has not been determined. To develop chromosome-specific landmarks, *A. cristatum* genomic DNA was sequenced, and new tandem repeats were discovered. Their distribution on mitotic chromosomes was studied by fluorescence in situ hybridization (FISH), which revealed specific patterns for five repeats in addition to 5S and 45S ribosomal DNA and rye subtelomeric repeats pSc119.2 and pSc200. FISH with one tandem repeat together with 45S rDNA enabled identification of all *A. cristatum* chromosomes. To analyze the structure and cross-species homoeology of *A. cristatum* chromosomes with wheat, probes for 45 mapped wheat cDNAs covering all seven chromosome groups were localized by FISH. Thirty-four cDNAs hybridized to homoeologous chromosomes of *A. cristatum*, nine hybridized to homoeologous and non-homoeologous chromosomes, and two hybridized to unique positions on non-homoeologous chromosomes. FISH using single-gene probes revealed that the wheat-*A. cristatum* collinearity was distorted, and important structural rearrangements were observed for chromosomes 2P, 4P, 5P, 6P and 7P. Chromosomal inversions were found for pericentric region of 4P and whole chromosome arm 6PL. Furthermore, reciprocal translocations between 2PS and 4PL were detected. These results provide new insights into the genome evolution within Triticeae and will facilitate the use of crested wheatgrass in alien gene introgression into wheat.

**Electronic supplementary material:**

The online version of this article (10.1007/s00122-018-3148-9) contains supplementary material, which is available to authorized users.

## Introduction

Among wild relatives of cultivated Triticeae, the genus *Agropyron* is a promising donor of important traits for improvement of bread wheat (*Triticum aestivum* L., 2n = 6x = 42, genome AABBDD). The most widely distributed species in the genus, *Agropyron cristatum* (L.) Gaertn (Yang et al. 2014), known as crested wheatgrass, deserves particular attention because of its high crossability with wheat and other Triticeae and because it is a source of a number of genes controlling resistance to biotic and abiotic stress, such as resistance to barley yellow dwarf virus (Sharma et al. 1984; Shukle et al. 1987), wheat streak mosaic virus (Sharma et al. 1984; Shukle et al. 1987; Brettell et al. 1988; Friebe et al. 1991), yellow rust, leaf rust and stem rust (Knott 1964, 1968; Cauderon and Rhind 1976), and powdery mildew (Wu et al. 2006; Luan et al. 2010; Han et al. 2014; Ye et al. 2015; Zhang et al. 2015; Song et al. 2016), as well as cold tolerance (Limin and Fowler 1990), salinity tolerance and drought tolerance (McGuire and Dvorak 1981), and genes affecting yield (Song et al. 2013).

As alien gene transfer by interspecific hybridization is affected by chromosome collinearity, it is important to establish syntenic relationships between chromosomes of the donor alien species and wheat (Friebe et al. 1996; Qi et al. 2007; Feuillet et al. 2008). If collinearity between the donor and recipient genomes is broken down due to evolutionary chromosome rearrangements, gene transfer by homoeologous chromosome recombination may result in progenies with non-balanced genomes (Devos et al. 1993; Zhang et al. 1998). Altered structures of the donor chromosomes may interfere with recombination and hamper attempts to reduce the size of introgressed chromatin and eliminate undesirable traits (Nasuda et al. 1998).

Crested wheatgrass is a perennial, self-compatible, facultative allogamous species comprising a complex of polyploid series based on the P genome: diploid (2*n *= 2*x *= 14, PP), tetraploid (*2n *= 4*x *= 28, PPPP), and hexaploid (2*n *= 6*x *= 42, PPPPPP) (Dewey 1983; Asay et al. 1992). The species is distributed from central Europe and the Middle East across Central Asia to Siberia, China, and Mongolia (Dewey and Asay 1982). To date, the knowledge of the *A. cristatum* genome remains poor and, as yet, the homoeology between *A. cristatum* and wheat chromosomes has not been investigated in detail. Only a few attempts were made to characterize the *A. cristatum* karyotype on the basis of chromosome morphology (Asghari et al. 2007; Yang et al. 2014). Han et al. (2014) found that the *A. cristatum* chromosome 6P differs from wheat homoeologs by large rearrangements. This observation underlines the need for detailed analysis of the structures of all *A. cristatum* chromosomes. The ability to identify *A. cristatum* chromosomes would open avenues for the detection of introgressed chromosomes and their segments in the genetic background of other Triticeae species.

Fluorescence in situ hybridization (FISH) is a widely used cytogenetic method to study chromosome structural alterations (Alkhimova et al. 1999; Benavente et al. 2008; Fu et al. 2013). The utility of FISH depends on the availability of probes that provide chromosome-specific labeling patterns. Only a few repetitive DNA sequences have been applied as FISH probes to *A. cristatum* chromosomes. Apart from chromosome-specific 45S and 5S ribosomal DNA repeats (Smit et al. 2007), Svitashev et al. (1998) isolated repeats pAgc1 (350 bp) and pAgc30 (458 bp) from *A. cristatum* and used them to identify the P genomes involved in the evolution of the *Elymus* species. Wu et al. (2010) cloned three *A. cristatum*-specific sequences and applied them to detect the P genome in wheat-*A. cristatum* addition lines. FISH with subtelomeric tandem repeats pSc119.2 and pSc200 isolated from rye (*Secale cereale* L.) (Bedbrook et al. 1980; Appels et al. 1981) was applied to *A. cristatum* (Badaeva et al. 1996, 2004; Schneider et al. 2005; Linc et al. 2017). To date, a standard FISH karyotype of *A. cristatum* on the basis of homoeologous relationships with other species from Triticeae has not been established. The lack of standard karyotype of *A. cristatum* hampers a wider use of chromosome mediated gene transfer into wheat.

While FISH with repetitive sequences has been useful in chromosome identification in numerous plant species (He et al. 2015; Tang et al. 2016), it is not reliable for detecting a cross-species homoeology (Danilova et al. 2014). The presence and copy number of repetitive elements varies among Triticeae species (Kato et al. 2004). Moreover, other types of probes, such as, telomeric, subtelomeric or centromeric probes do not permit a complete identification of all chromosomes and will not facilitate a precise comparative analysis in most species of Triticeae (Tang et al. 2008, 2014; Mirzaghaderi et al. 2014; Fu et al. 2015). In contrast, genic sequences and their order are generally more conserved in grass genomes (Gale and Devos 1998). Devos and Gale (2000) and Paterson et al. (2000), utilized the existence of conserved sequences, usually cDNAs, to study the homoeologous relationship between related species from Triticeae.

Visualization of single-genes on mitotic metaphase chromosomes allows to investigate chromosome rearrangements and cross-species homoeology (Danilova et al. 2012, 2014, 2017; Karafiátová et al. 2013). To visualize low-copy sequences or small genomic targets on chromosomes, different types of probes have been used. Jiang et al. (1995) and Kim et al. (2005) used genomic BAC clones as probes for physical mapping of rice and sorghum genomes. This strategy works successfully in plant species with small gene-rich genomes. In plant species with bigger genomes, the application of cDNA sequences as FISH probes allowed chromosome identification, comparative analysis of chromosome structure between species, and identification of gene order along the chromosomes. The method called single-gene FISH was successfully used in rice (Kharb et al. 2001), maize (Wang et al. 2006), barley (Karafiátová et al. 2013), wheat and its wild relatives; i.e., *Aegilops caudata*, *Ae. markgrafii,* and *Ae. umbellulata* (Danilova et al. 2014, 2017). Danilova et al. (2014, 2017) used wheat cDNA sequences from Kawaura et al. (2009) as FISH probes to develop a wheat single-gene FISH map and analyze homoeologous relationships and chromosomal rearrangements within the Triticeae. Development of a single-gene FISH map for *A. cristatum* could help in chromosome identification and study the evolutionary genome rearrangements of this species in relation to wheat.

The objective of the present study was to develop a molecular karyotype and analyze the homoeology of the diploid *A. cristatum* chromosomes with those of bread wheat. During the course of the work, P genome-specific repetitive sequences were isolated, and their chromosome distribution patterns were explored. A set of wheat cDNA sequences were localized on *A. cristatum* chromosomes, and the cross-genome homoeology was established.

## Materials and methods

### Plant material and genomic DNA isolation

The seeds of diploid *A. cristatum* cv. Parkway (2*n *= 2*x *= 14, PP), accession number PI 415799, released by the Canada Department of Agriculture in 1969, were kindly provided by Dr. Joseph Robins, ARS Forage and Range Research Laboratory, USDA, Logan, USA. The seeds were germinated on moistened filter paper in a Petri dish and transferred to the soil. Plants were grown in a greenhouse, and genomic DNA was isolated from young leaf tissues using the NucleoSpin Plant II kit (Macherey–Nagel GmbH & Co. KG, Düren, Germany) following the manufacturer’s recommendations. DNA content analysis was performed according to Doležel et al. (2007) using *Secale cereale* cv. Dankovske (2C = 16.19 pg DNA) as internal reference standard.

### Illumina sequencing

Libraries for sequencing were prepared from genomic DNA using the TruSeq DNA PCR-Free Library Preparation Kits (Illumina, San Diego, USA). Two micrograms of DNA dissolved in 100 μl of deionized water were fragmented with Bioruptor Plus (Diagenode, Denville, USA) with the following settings: 5 cycles, 30/90 (On/Off) seconds. DNA was sheared to 500–700-bp fragments, purified, end-repaired, adenylated, size-selected to 1000 bp and ligated with adapters according to the manufacturer’s protocol (Illumina). Libraries were diluted 1:10,000, and their concentration was estimated by real-time PCR using the KAPA Library Quantification Kit (Kapa Biosystems, Woburn, USA). The libraries were then pooled and diluted to a final concentration of 17 pM. Samples were paired-end sequenced on an Illumina MiSeq instrument for 600 cycles using the MiSeq Reagent Kit v3 (Illumina).

### Sequence data analysis and identification of tandem organized repeats

Illumina reads were trimmed for adapters and for quality using the FASTX-toolkit [-q 20 -p 90] (http://hannonlab.cshl.edu/fastx_toolkit/index.html). Chloroplast DNA sequences were identified and filtered out from both datasets prior to further analysis using the ERNE-FILTER program (Del Fabbro et al. 2013), and then, a stand-alone version of the Repeat Explorer pipeline (Novák et al. 2013) was used for de novo repeat identification and characterization. In the first step, a data set of 1,393,600 250-bp long paired-end reads were randomly selected and used for the reconstruction of repetitive DNA elements using the graph-based method according to Novák et al. (2010). This dataset represented 0.05 × coverage of the *A. cristatum* genome. Characterization and annotation of the assembled sequences were completed using similarity searches by *RepeatMasker* (http://www.repeatmasker.org) and its Viridiplantae databases, which were augmented with other repeats of selected plant species (Novák et al. 2010), and by BLASTX and BLASTN (Altschul et al. 1990) against the GenBank NR (Benson et al. 2010) and protein database created specifically for the Repeat explorer pipeline (Novák et al. 2010). All putative tandem organized repeats were identified using Dotter (Sonnhammer and Durbin 1995), and contigs with tandem repeats were then used for specific primer design using the Primer3 program (Untergasser et al. 2012). Newly characterized tandem repeats of *A. cristatum* were deposited in the GenBank/EMBL data libraries under accession numbers MG323511–MG323515.

### Preparation of cDNA probes

We selected 45 cDNA probes previously mapped by single-gene FISH to bread wheat chromosomes by Danilova et al. (2014). The cDNA clones were developed by the National BioResource Project-Wheat, Japan. One sequence; the *Acc2* cDNA pooled probe included in this study was developed from wheat RNA by Danilova et al. (2012). All cDNA clones were kindly supplied by the Department of Plant Pathology, Wheat Genetics Resource Center, Kansas State University. cDNA sequences were amplified using PCR with T3/T7 primers and LongAmp DNA Polymerase (New England Biolabs, Massachusetts, USA) following the manufacturer’s recommendations. The PCR products were purified with the Invitrogen PCR purification kit (Life Technologies, Carlsbad, USA) according to the manufacturer’s instructions.

### Probe labeling

Probes for newly identified tandem repeats were labeled by PCR either with biotin-dUTP or digoxigenin-dUTP (Roche, Mannheim, Germany) using primers listed in Table S1 and *A. cristatum* DNA as a template. The PCR mix contained 30 ng of genomic DNA, reaction buffer (consisting of 10 mM Tris–HCl pH 8, 50 mM KCl, 0.1% Triton X-100 and 1.5 mM MgCl_2_), 200 μM dNTPs including biotin-dUTP or digoxigenin-dUTP, 1 μM primers and 0.4 U of Taq polymerase (New England Biolabs). The thermocycler conditions were set as follows: initial denaturation step at 94 °C for 5 min, followed by 35 cycles of denaturation (94 °C/50 s), annealing at 54 °C for 50 s and extension (72 °C/50 s). The final extension was allowed for 5 min at 72 °C. Probes for rye pSc119.2 and pSc200 repeats (Bedbrook et al. 1980 and Appels et al. 1981) were labeled with biotin-dUTP. The 120-bp unit of pSc119.2 inserted into the plasmid pBR322 was amplified by PCR using the M13 universal primers following Nagaki et al. (1995) and Contento et al. (2005). Genomic rye DNA was used as a template to amplify pSc200 by PCR according to Tang et al. (2008).

Probes for rDNA sequence were labeled either with biotin-dUTP, digoxigenin-dUTP, or with a fluorochrome. In all cases, template rice DNA for the 5S rDNA probe was amplified by PCR according to Fukui et al. (1994), while the plasmid pTa71 (45S rDNA) containing 9-kb fragment from *T. aestivum* with 18S-5.8S-26S rDNA and intergenic spacers (Gerlach and Bedbrook 1979) was labeled by nick translation following Kato et al. (2004, 2006). The 45S rDNA was directly labeled with ChromaTide^®^ Alexa Fluor^®^ 546-14-dUTP (Thermo Fisher Scientific, Waltham, USA), while 5S rDNA was directly labeled with ChromaTide^®^ Fluorescein-12-dUTP (Thermo Fisher Scientific). Out of the newly identified tandem repeats, ACRI_CL78 was also directly labeled with ChromaTide^®^ Fluorescein-12-dUTP (Thermo Fisher Scientific).

All cDNA probes were labeled with ChromaTide^®^ Texas Red^®^-12-dUTP (Thermo Fisher Scientific). Direct probe labeling for cDNA and 45S rDNA by nick translation using 2 μg DNA was done as described by Kato et al. (2004, 2006). The probe quality was checked on 1.5% agarose gel. To increase probe concentration and remove non-incorporated nucleotides, probes were precipitated and purified by adding 173 μl 1 × TE buffer (pH 7.5), 20 μl NaAc (3 M, pH 5.2) and 500 μl 100% ethanol; 3 μg herring sperm DNA (Promega, Wisconsin, USA) was added to block DNA. After overnight precipitation at − 20 °C, the probes were centrifuged for 30 min at 4 °C at 14,000 × *g*, rinsed in 70% ethanol and air-dried for 7–15 min. Subsequently, probes were dissolved in 20 μl of 2 × SSC and 1 × TE buffer (pH 7.6) in a 1:1 ratio at 65 °C for 10 min.

### Mitotic chromosome preparations

Seeds of *A. cristatum* were germinated on moistened filter paper in a glass Petri dish in the dark at 25 °C for 3–4 days. Root tips were transferred to distilled water and incubated overnight at 1 °C in a box filled with ice-water. Subsequently, the root tips were fixed in ice-cold 90% acetic acid for 10 min followed by 3 washes in 70% ethanol and stored in 70% ethanol at − 20 °C. Chromosome preparations were prepared using the drop technique according to Kato et al. (2004, 2006), with minor modifications as described in Danilova et al. (2012). The quality of chromosome spreads was checked under a microscope, and the best slides were selected for FISH.

### Fluorescence in situ hybridization (FISH)

All FISH experiments were done with two or more labeled probes which were hybridized simultaneously. New repetitive DNA sequences, pSc119.2, pSc200 and rDNA were localized following the protocol of Cabrera et al. (2002) with modifications. Briefly, digoxigenin-labeled probes were detected using anti-digoxigenin fluorescein isothiocyanate (Roche). Biotin-labeled probes were detected with Cy3-conjugated streptavidin (Invitrogen). Hybridization mixture per slide (total volume = 10 μl) contained 50 ng labeled probe DNA, 50% v/v formamide, 2 × SSC (0.15 mol/l NaCl plus 0.015 mol/l sodium citrate), 10% w/v dextran sulfate, 0.4 μg salmon sperm DNA and 0.1% w/v sodium dodecyl sulfate. Low-copy sequences were localized in combination with 45S rDNA and one tandem repeat (ACRI_CL78) or 5S rDNA by FISH following Karafiátová et al. (2013) with minor modifications. Hybridization mixture (20 μl) consisting of 10 μl formamide, 2 × SSC, 0.1 M Tris–HCl pH 8, 0.05 M EDTA, 1 μg/μl salmon sperm DNA, 300 ng cDNA probe, 50 ng DNA for 45S rDNA and 50 ng of tandem repeat (ACRI_CL78) or 5S rDNA applied onto slides, and the cover slides were taped up. The chromosomes and probes were denatured together at 80 °C for 3 min under high moisture conditions. The hybridization was carried out overnight at 37 ºC; then, the slides were washed in 2 × SSC for 1 min at RT, 2 × SSC for 20 min at 55 °C and 2 × SSC for 1 min at RT, followed by dehydration in 70, 90, 100% ethanol, with each step lasting 2 min. The chromosomes were mounted and counterstained with 4′,6-diamidino-2-phenylindole (DAPI) in Vectashield media (Vector Laboratories, Burlingame, USA).

### Microscopy, software, signal capture and image analysis

Chromosome preparations were examined using an Axio Imager Z.2 Zeiss microscope (Zeiss, Oberkochen, Germany) equipped with a Cool Cube 1 (Metasystems, Altlussheim, Germany) camera and appropriate filter sets. The signal capturing and picture processing were performed using ISIS software (Metasystems). The final image adjustment was done in Adobe Photoshop CS5 (Adobe Systems Incorporated, San Jose, USA). Chromosome measurements and determination of the position of FISH signals were measured using ISIS software (Metasystems).

### Chromosome measurements

To establish the karyotype and idiogram, ten best images of metaphase spreads obtained at 100× magnification were selected and mean chromosome arm lengths were determined in micrometers (μm). The centromeric index was calculated by dividing the length of the shorter of the two chromosome arms by the length of the whole chromosome and expressed in percent. Arm ratio was calculated by dividing the length of the longer arm of the chromosome by the length of the shorter arm. Relative length was determined by dividing the length of a particular chromosome by the total length (H) of chromosomes in the haploid set and expressed in per cent (Table S2). Individual chromosomes were classified according to centromeric index. The relative positions of cDNA FISH sites were measured from the centromere. The positions given are average values determined from five measurements on different metaphase spreads. The average relative distance from the centromere, standard deviation and confidence intervals with a significance level of 0.05 were calculated using Microsoft Office Excel 2010 functions. The cDNA positions are summarized in Table S3. The cDNA positions on wheat chromosomes and the wheat idiogram were drawn according to the data from Gill et al. (1991) and Danilova et al. (2014).

## Accession numbers

Plant material; diploid *A. cristatum* cv. Parkway (2*n* = 2*x* = 14, PP), accession number PI 415799. Newly characterized tandem repeats of *A. cristatum* can be found in the GenBank/EMBL data libraries under accession numbers MG323511–MG323515.

## Results

### Identification and genomic distribution of tandem repeats

#### 5S and 45S rDNA

FISH with a probe for 45S rDNA showed four hybridization sites on the short arm of two chromosome pairs corresponding to two pairs of nucleolus organizing regions (NOR). The 5S rDNA probe showed signals in a subterminal position of the short arms of one of the chromosome pairs carrying 45S rDNA locus. Later, this chromosome pair was identified based on cDNA mapping as 5P, while the other pair was identified as 1P, which is characterized by secondary constriction and a satellite on the short arm. Interestingly, another chromosome with a secondary constriction and satellite on the short arm was observed but had no 5S or 45S rDNA signals (Supplementary Fig. S1). Using cDNA probes from wheat, this chromosome pair was identified as 2P. Thus, two satellite chromosome pairs (1P and 2P) were characterized by secondary constriction and satellites on the short arm, but only 1P corresponds to the NOR position confirmed by the 45S rDNA signal. The chromosome 5P carried 45S and 5S signals on terminal and subterminal positions, respectively. The use of 5S and 45S rDNA probes simultaneously enabled identification of two out of the seven pairs of *A. cristatum* chromosomes.

#### The pSc119.2 and pSc200 sequences

**Fig. 1:**
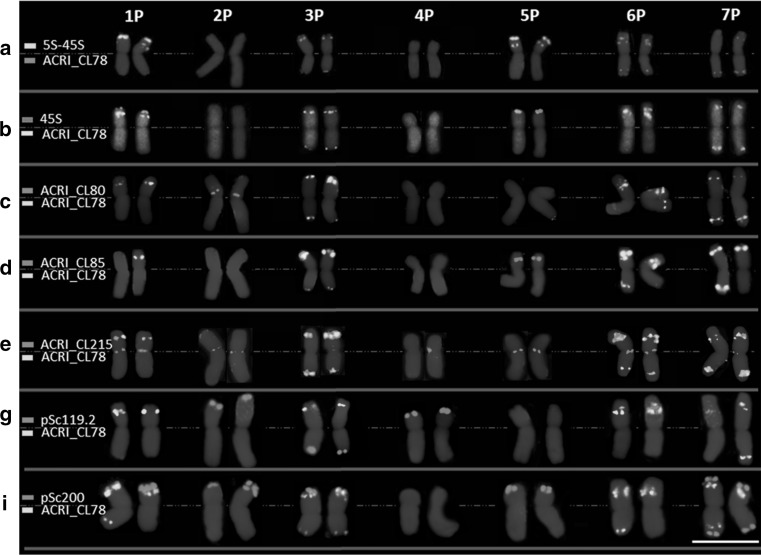
FISH on mitotic chromosomes of diploid *A. cristatum* cv. Parkway with probes for 5S and 45S rDNA, newly identified tandem repeats ACRI_CL78, ACRI_CL80, ACRI_CL85 and ACRI_CL215, and rye tandem repeats pSc119.2 and pSc200. Bar = 10 μm

**Fig. 2:**
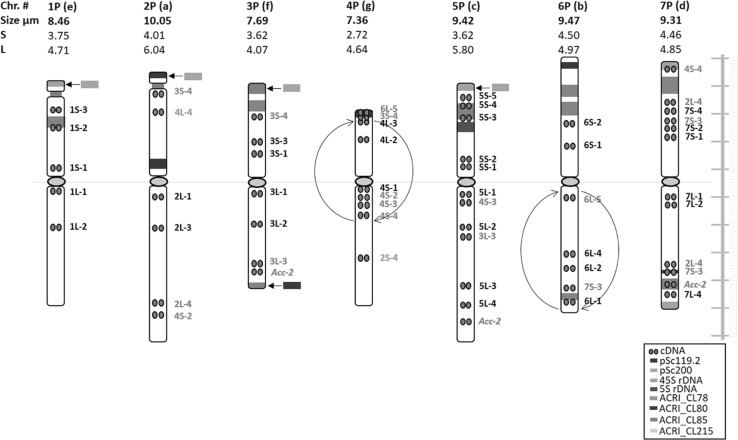
Idiogram for chromosomes of diploid *A. cristatum* cv. Parkway showing the distribution of 5S and 45S rDNA gene clusters, newly identified tandem repeats ACRI_CL78, ACRI_CL80, ACRI_CL85 and ACRI_CL215, rye tandem repeats pSc119.2, pSc200, and cDNAs. The names of cDNA probes that hybridized to more than one chromosome are highlighted in red. The names of cDNA probes that hybridized only to a non-homoeologous chromosome are highlighted in green. Inversions on chromosomes 4P and 6PL are indicated by circular arrows. The letters in parentheses show the order of the chromosome by size from the longest (a) to the shortest (g). S and L letters refer to the short and long arm, respectively. The color scheme (bottom right) shows the color of each probe as represented in this idiogram. The scale on the right side, consists of 10 units, and each unit represents 1 μm

Three of the seven chromosome pairs possessed pSc119.2 repeat clusters. Chromosomes 2P and 4P carried pSc119.2 signals on short arms, while 3P carried them on the long arm. Five chromosome pairs carried pSc200 signals. FISH with the pSc200 probe gave FISH signals on the short arms of chromosomes 1P, 2P, 3P and 5P, while on 7P, the signals were on both short and long arms. In all cases, pSc119.2 and pSc200 probes gave strong telomeric signals (Figs. 1 and 2, and Supplementary Fig. S2). Two chromosomes, 3P and 7P, could be distinguished from the remaining *A. cristatum* chromosomes by characteristic signals of pSc119.2 and pSc200, respectively. The chromosome 3P was identified by a unique and strong signal of pSc119.2 on the tip of the long arm, while 7P was characterized by clear pSc200 signals on the terminal regions of both arms.

#### New tandem repeats isolated from *A. cristatum*

Using flow cytometry, we estimated 2C DNA amount of 12.99 ± 0.11 pg DNA (mean ± SD) for *A. cristatum*. Its 1C genome size was determined as 6352 Mbp considering 1 pg DNA = 0.978 Mbp (Doležel et al. 2003). After quality filtering and trimming, the sequences to 250 bp, partial Illumina sequencing of *A. cristatum* resulted in a cumulative length of ~ 7639 Mb, corresponding to ~ 1x coverage of the nuclear genome. A set of randomly selected paired-end reads representing 0.05× genome coverage was used for repeat reconstruction and characterization using the Repeat Explorer pipeline. In general, Ty3/*gypsy* elements (38.10%) were found to be more abundant than Ty1/*copia* (18.57%), while non-LTR elements and DNA transposons accounted for only 0.15 and 3.28% of the genome, respectively (Table 1). Among the Ty3/*gypsy* superfamily, the elements from the Athila lineage were the most common and one particular element of the Athila lineage accounted for more than 20% of the nuclear genome. The Ty1/*copia* superfamily was mostly represented by retroelements from the Angela lineage (~ 13.5%) and the Sire/Maximus lineage (~ 4.4%). The elements representing other lineages of Ty1/*copia* were rare (Table 1). Dotter analysis was applied to all assembled contigs from clusters with layouts indicating tandem characteristics (Novák et al. 2010) and revealed fourteen different types of tandem repeats in *A. cristatum*. The most abundant repeat, ACRI_CL20, had a 379-bp long unit and comprised ~ 0.48% of the nuclear genome. A comparison of ACRI_CL20 and the pSc200 satellite (GenBank accession number Z54189) revealed 93% sequence identity. Two other tandem repeats showed similarities to known plant satellites; ACRI_CL78 was 80% similar to the tandem repeat 4P6 (AY249985) from *Aegilops tauschii* Coss. (2*n *= 2*x *= 14, genome DD) (Zhang et al. 2004). Tandem repeat ACRI_CL215 had 87% similarity to tandem repeat AB016970 of the pLrTail family, first characterized in *Leymus racemosus* (GB code: AB016970). BLAST searches with 9 putative tandemly organized repeats identified in *A. cristatum* revealed homology to some parts (usually non-annotated) of wheat 3B chromosome pseudomolecule, and two repeats (ACRI_CL85 and ACRI_CL198) did not show any homology with sequences deposited in the GenBank database.

**Table 1:** Proportion of repetitive DNA sequences of *A. cristatum* cv. Parkway identified after partial Illumina sequencing

Repeat		Lineage/class	Alternative names	Proportion in the analyzed data [%]
LTR retroelements	*Ty1/copia*	Maximus-SIRE		4.42
		Angela		13.51
		TAR	Tont	0.56
		Tork	Tnt	0.01
		Ale	Hopscotch	0.05
		Bianca		0.02
		Total *Ty1/copia*		18.57
	*Ty3/gypsy*	Athila		22.96
		Chromovirideae		7.16
		Ogre-Tat		7.98
		Total *Ty3/gypsy*		38.10
Other	LINE			0.15
	DNA transposons			3.28
	Tandem repeats			1.62
	rRNA genes			0.21
Unclassified LTR elements				3.94
Unclassified repeats				6.71
Non-annotated sequences				8.36

Out of the fourteen tandem repeats, eleven were used as FISH probes, and five of them showed specific distribution patterns. For one of them (ACRI_CL187), dispersed labeling was observed on all chromosomes, except in centromeric and most pericentromeric regions. Repeat ACRI_CL215 showed a preferential location in primary constrictions of all chromosomes. FISH with a probe for the ACRI_CL78 repeat identified the seven pairs of chromosomes, and it is thus recommended for karyotyping. Although there was a slight difference in the signals pattern of the repeat sequence ACRI_CL78 between the homologous chromosomes from different plants of the diploid *A. cristatum*. However, these small polymorphisms did not hamper the precise characterization of the individual chromosomes. Two tandem repeats gave a fewer number of FISH signals, and each of them showed characteristic patterns on two different chromosome pairs; for instance, ACRI_CL80 showed a strong signal on the pericentromeric region of 2PS and the subterminal region of 6PS, while ACRI_CL85 distinguished other two chromosomes by clear signals on the terminal region of the short arm of chromosome 3P and the subterminal region of the short arm of 5P. (Figs. 1 and 2, and Supplementary Fig. S2). The five new tandem repeats enabled the precise characterization of all the individual chromosomes; the labeling patterns were always chromosome specific. The remaining six new tandem repeats did not show any FISH signals on chromosomes of *A. cristatum*.

#### Karyotype analysis

The measurement and chromosome classification is summarized in Supplementary Table S2. All examined mitotic metaphase plates showed standard diploid constitution (2*n* = 14), characterized by seven pairs of chromosomes, two pairs being satellite chromosomes. During the preparation of the karyotype, the homologous chromosomes were tentatively paired based on tandem repeat ACRI_CL78 distribution, morphology, size, and the presence of 5S and 45S rDNA loci. In the karyotype, each chromosome was identified by a specific number and small letter (Fig. 2). The numbers ranged from 1 to 7, indicating the correspondence of each chromosome to its homoeolog in bread wheat according to the cDNA data. The letters differed between ‘a’ and ‘g’. The letter ‘a’ indicates the largest chromosome, while the letter ‘g’ indicates the smallest one. The chromosome sizes ranged from 7.36 to 10.05 μm. The relative *A. cristatum* chromosome length ranged from 16.27% for the largest chromosome, 2P, to 11.92% for the smallest, 4P (Supplementary Table S2). Following Naranjo et al. (1983) and based on the data in Supplementary Table S2, the chromosomes of *A. cristatum* were classified into two different types based on centromere position and arm ratio. Four chromosomes were metacentric (1P, 3P, 6P and 7P), while the rest were submetacentric (Fig. 2). Identification of the *A. cristatum* chromosomes using FISH pattern of the tandem repeat ACRI_CL78 together with the chromosome size and centromere position, facilitated the co-localization of the remaining tandem repeats and further mapping of cDNAs (Fig. 1 and 2 and Supplementary Fig. S2). The results are summarized in the idiogram representing chromosomes, arranged according to their homoeology to wheat based on cDNA data (Fig. 2).

#### Chromosome structure and rearrangements as revealed by cDNA FISH

**Fig. 3:**
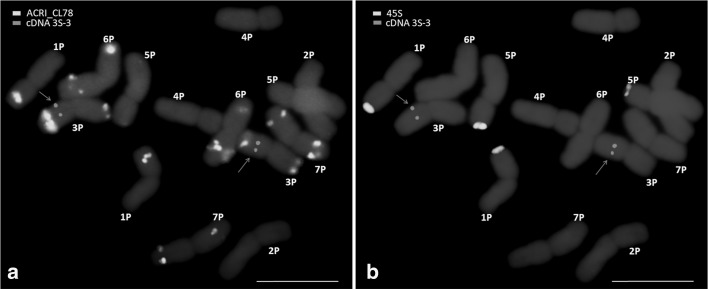
FISH on mitotic chromosomes of diploid *A. cristatum* cv. Parkway with probes for the tandem repeat ACRI_CL78, cDNAs (red arrows) and 45S rDNA. **a** Tandem repeat ACRI_CL78 (green) and cDNAs (red); **b** 45S rDNA (green) and cDNAs (red). The chromosomes were counterstained with DAPI (blue). Bars = 10 μm

A set of 45 cDNA probes which were mapped to bread wheat chromosomes (Danilova et al. 2014) was used. FISH with the cDNA probes produced clear signals on chromosomes of *A. cristatum* (Fig. 3). The forty-five cDNA probes hybridized to fifty-eight positions on *A. cristatum* chromosomes (Fig. 2 and Supplementary Fig. S3, and Supplementary Table S4). Apart from the hybridization of thirty-four probes (75.6%) to homoeologous chromosomes on thirty-four positions, nine (20%) probes hybridized to non-homoeologous chromosomes of *A. cristatum* on twenty-two positions: probe 2L-4 hybridized to chromosome arms 2PL, 7PS and 7PL; probe 3S-4 hybridized to 2PS, 3PS and 4PS; probe 3L-3 hybridized to 3PL and 5PL; probe *Acc*-*2* hybridized to 3PL, 5PL and 7PL; probe 4S-2 hybridized to 2PL and 4PL; probe 4S-3 hybridized to 4PL and 5PL; probe 4S-4 hybridized to 4PL and 7PS; probe 6L-5 hybridized to 4PS and 6PL; and probe 7S-3 hybridized to 6PL, 7PS and 7PL. Some of the cDNA probes hybridized to more than one position per chromosome. For example, probes 2L-4 and 7S-3 produced signals on both arms of chromosome 7P, such case was not reported in wheat. While the number of the cDNA probes located on multiple loci (A*cc*-2, 2L-4, 3S-4, 3L-3, 4S-2, 4S-3, 4S-4, 6L-5, 7S-3) on *A. cristatum* genome was higher, and representing 20% of the used cDNAs, in wheat genomes, only about 7% of these cDNAs (*Acc*-*2*, 4S-3 and 4S-4) were located on more than one locus, on chromosome arms 3L, 4AL, and 5L; 4S, 4AL, 5L, and 7AL; 2S, 4S and 4AL, respectively. Besides the hybridization of the forty-three cDNA probes described here, two (4.4%) cDNA probes (2S-4 and 4L-4) hybridized to unique sites on non-homoeologous chromosomes (4PL and 2PS, respectively) of *A. cristatum*.

A comparison between the cDNA positions on diploid *A. cristatum* cv. Parkway and the homoeologous groups of hexaploid wheat cv. Chinese Spring are shown in Supplementary Fig. S4. Most of the probes hybridized to corresponding homoeologous chromosome arms of *A. cristatum*, generally in the same order, indicating preserved cross-genome collinearity. No major collinearity distortion was detected on chromosomes 1P, 3P and 5P, though additional probes need to be applied to 1PL to verify its structure. However, many cDNA hybridization sites indicated the collinearity distortion for the rest of the *A. cristatum* genome. For example, signals of cDNA probes 2S-4 on 4PL and probe 4L-4 on 2PS indicate reciprocal translocation between chromosome arms 2PS and 4PL. Moreover, all of the group-4 short arm-specific cDNA probes hybridized to the long arm, while all of the group-4 long arm-specific cDNA probes hybridized to the short arm, thus suggesting pericentric inversion on 4P. On the long arm of chromosome 6P, the probes hybridized in an inverted order as compared to their order on homoeologous wheat chromosome arm, which points to a paracentric inversion of 6PL. The presence of signals of nine cDNAs in multiple loci may indicate either a gene or a chromosome segment duplication.

## Discussion

### Secondary constrictions and nucleolus organizing regions (NORs)

We found four 45S rDNA sites and two 5S rDNA site in the diploid chromosome set of *A. cristatum*. The 45S sites were located at the terminal position of the short arms of 1P and 5P, 1P carry a secondary constriction, while on 5P no secondary constriction was observed. The chromosome 2P carries a secondary constriction, but the 45S rDNA signals were absent. Two pairs of unidentified satellite chromosomes in *A. cristatum* were observed in previous studies (Hsiao et al. 1986, 1989). Linc et al. (2017) observed one chromosome pair with a clear secondary constriction in two accessions of diploid *A. cristatum*, but the constriction on the second chromosome pair was visible only in the genotype MvGB1521. Moreover, they reported that two chromosome pairs carrying 45S signals on the short arms were found in one accession MvGB1521, but in the accession MvGB1509, the signals were detected only on one chromosome pair, while the other chromosome pair showed pSc119.2 site at the short arm instead. The 45S rDNA on the terminal position of the *A. cristatum* chromosome arm 5PS was detected by Jubault et al. (2006) in the wheat-*A. cristatum* chromosome addition line. Secondary constrictions or NOR sites are commonly located in the terminal regions of the chromosomes (Sousa et al. 2011). In many cases, the 45S rDNA sites were observed to be restricted to the NORs, although in various species other smaller or less active sites have also been detected outside the NORs (Sato et al. 1980; Guerra et al. 1996; De Carvalho and Guerra 2002). Our FISH results for chromosome 1P and 5P are in agreement with these observations. However, it seems that secondary constrictions in this species are not always related to the rDNA unit, and this observation deserves further investigation.

To date, there was no information about genomic distribution of 5S rDNA sites in *A. cristatum*. We located 5S rDNA locus in subterminal region of the short arm of chromosome 5P. In most Triticeae, the 5S rDNA sites are located in homoeologous groups 1 and 5 (Dvorák et al. 1989; Reddy and Appels 1989). In *Thinopyrum ponticum*, which is related to *A. cristatum*, the 45S rDNA locus is distally located in relation to the 5S rDNA locus (Brasileiro-Vidal et al. 2003). This disposition is similar to our results on 5P and to that found in homoeologous group 5 of the D genome of wheat (Mukai et al. 1990, 1991) and *Aegilops umbellulata* (Castilho and Heslop-Harrison 1995).

### Chromosome morphology

Relative chromosome length can be used as a rough estimate of the proportion of the genome that a given chromosome represents (Jabeen et al. 2012). We found that chromosome 2P is the longest, while 4P is the shortest chromosome in the karyotype of *A. cristatum* (Table S2). The data on chromosome size are of special importance if these chromosomes are intended to be sequenced. We observed that four chromosomes of diploid *A. cristatum* were metacentric, while the rest were submetacentric, which is in agreement with data of Linc et al. (2017). In contrast, Yang et al. (2014) found karyotypes of *A. cristatum* from several populations to be variable, as the chromosomes of some diploids were all metacentric, while other di-, tetra- and hexaploids showed a combination of metacentric and submetacentric chromosomes. We compared the size of *A. cristatum* chromosomes with those of wheat in Gill et al. (1991). The chromosomes of *A. cristatum* were in general shorter than their homoeologous chromosome groups from bread wheat (Supplementary Fig. S4). The chromosome morphology of *A. cristatum* is of special importance for genomic studies and the quick identification of alien chromosomes in the substitution or addition lines.

### FISH pattern of tandem repeats allows identification of all *A. cristatum* chromosomes

With the aim to achieve reliable identification of all *A. cristatum* chromosomes, we determined chromosome distribution of two tandem repeats pSc119.2 and pSc200. Linc et al. (2017) performed FISH with the same repeats in two *A. cristatum* accessions and found that one of the two accessions (MvGB1509) showed the same number of pSc119.2 signals as we found on *A. cristatum* cv. Parkway. Previous studies on Triticeae showed that combination of pSc119.2 and other FISH probes in most species does not permit identification of all chromosomes (Mirzaghaderi et al. 2014; Li et al. 2018). These tandem repeats were present in genomes of several species forming large telomeric blocks, especially in genera *Aegilops* and *Secale,* and were used to distinguish between their chromosomes (Tang et al. 2008, 2014; Mirzaghaderi et al. 2014; Fu et al. 2015). In bread wheat, the pSc119.2 probes hybridized to B genome chromosomes in addition to chromosomes 4A, 5A, 2D, 3D, and 4D, while the signals of pSc200 were not observed (Tang et al. 2008, 2014; Fu et al. 2015). However, pSc200 presented 93% sequence identity to ACRI_CL20, the most abundant tandem repeat in the *A. cristatum* genome, and was similar to two other tandem repeats. After comparing hybridization patterns of pSc119.2 and pSc200 in *A. cristatum* as observed in our study and in wheat (Schneider et al. 2003; Contento et al. 2005; Tang et al. 2008, 2014; Fu et al. 2015), we concluded that each of the two probes produced characteristic patterns on chromosomes of *A. cristatum*, but only the use of pSc200 facilitates detection of the P genome in wheat introgression lines. For instance, using pSc200, five chromosomes (1P, 2P, 3P, 5P and 7P) could be distinguished. Two chromosomes 3P and 7P can be easily distinguished from the rest of *A. cristatum* chromosomes based on the pattern of pSc119.2 and pSc200 hybridization.

To distinguish all *A. cristatum* chromosomes using FISH, new tandem repeats were needed. Han et al. (2017) found that some *A. cristatum* repetitive sequences were distributed all over the P genome or at centromeres, pericentromeric regions, distal regions, and terminal regions. The sequences located at telomeres or subtelomeres were mostly tandem repeats, similar to pSc200 and pSc250 from rye (Vershinin et al. 1995), pAesKB52 from *Ae. speltoides* (Anathawat-Jonsson and Heslop-Harrison 1993), and pHvMWG2315 from barley (Busch et al. 1995). Although the sequences of Han et al. (2017) enabled identification of wheat-*A. cristatum* introgression lines, they did not permit identification of individual *A. cristatum* chromosomes. We obtained sequences of tandem repeats located at centromeric, telomeric, subtelomeric and pericentric regions by whole-genome sequencing, which were found to give specific patterns for individual *A. cristatum* chromosomes and enabled discrimination among all of the chromosomes. In addition, the region-specific repeats could be helpful in deducing the part of a P genome chromosome in wheat-*A. cristatum* translocation and introgression lines, as was shown by Li et al. (2012) for *Dasypyrum villosum* and Han et al. (2017) for *A. cristatum* introgression lines.

In our work, five tandem repeat sequences with different distribution patterns on the *A. cristatum* genome were identified by FISH. We found that the distribution patterns as well as the signal intensity of the tandem repeats were specific to different chromosomes. Thus, *A. cristatum* chromosomes can be identified using the hybridization pattern together with chromosome size and arm ratio. In addition, tandem repeats could be used to study the chromosome rearrangements during polyploidization (Han et al. 2005, 2017; Zhang et al. 2013). Hence, future studies on differences in genomic distribution of specific repetitive sequences between several populations from *A. cristatum* could be used to reveal their different roles during *A. cristatum* genome evolution (Han et al. 2017). The absence of FISH signals for six out of the eleven tandem repeats could be attributed to the fact that these sequences do not form clusters long enough to be detected by fluorescence microscopy. Similar findings were reported by Han et al. (2017), who isolated repetitive sequences from chromosome 6PS of tetraploid *A. cristatum* and only fourteen out of 48 sequences were detectable by FISH. In the present work, the slight differences observed in the FISH pattern of the repeat ACRI_CL78 between the homologous chromosomes of different plants from the same genotype can be attributed to the open pollinated nature of *A. cristatum*. This phenomenon was also reported for other open pollinated species such as rye (Szakács and Molnár-Láng 2008) as well as in other genotypes of diploid *A. cristatum* (Linc et al. 2017).

### cDNA revealed chromosome structure and evolutionary rearrangements in the P genome

The fact that a considerable percentage (24.4%) of the wheat cDNA sequences localized to both homoeologous and non-homoeologous chromosomes and that other mapped to non-homoeologous chromosomes indicates that some *A. cristatum* chromosomes did not preserve cross-species collinearity. Distorted cross-species collinearity was observed in chromosomes 2P, 4P, 5P, 6P and 7P. The order of cDNA probes in the pericentric region of chromosome 4P is inverted as compared to homoeologous regions of wheat chromosomes 4B and 4D shown in Danilova et al. (2014), but in accordance with their order on the homoeologous region of 4A. Chromosome 4A of wheat is known to be rearranged as a result of a 4AL/5AL translocation followed by a 7BS translocation and one paracentric and two pericentric inversions (Naranjo et al. 1987; Devos et al. 1995; Mickelsonyoung et al. 1995; Miftahudin et al. 2004). Thus, the *A. cristatum* chromosome 4P underwent paracentric inversion similar to wheat 4A. The inverted order of cDNA probes on the long arm of chromosome 6P when compared to their order on homoeologous wheat chromosomes (Danilova et al. 2014) indicates the presence of a P genome-specific paracentric inversion. A similar observation on chromosome 6P from the tetraploid *A. cristatum* was made by Han et al. (2014). This phenomenon of rearrangements can be found in the genomes of wild relatives of wheat (Kishii et al. 2004; Said and Cabrera 2009; Wang et al. 2010; Wang 2011; Hu et al. 2012; McArthur et al. 2012; Said et al. 2012; Han et al. 2014). Although our data showed chromosomal rearrangements in *A. cristatum* with respect to wheat, it seems that this does not hamper alien chromosome introgression into wheat. For instance, a series of disomic addition and translocation lines have been obtained after hybridization of bread wheat with *A. cristatum* and have shown high resistance to powdery mildew and leaf rust, longer grain length and higher thousand-grain weight (Li et al. 1998; Song et al. 2013; Ochoa et al. 2015; Li et al. 2016).

Transferring desirable alien genes and improving their utilization efficiency in wheat improvement can be hampered by linkage drag and insufficient compensation for the substituted wheat chromatin. To overcome this difficulty, it is necessary to identify the alien chromosomal regions of target genes and to analyze their homoeologous relationships with wheat chromosomes. Only well-compensating translocations or introgressions produced by induced homoeologous recombination are beneficial for wheat improvement (Qi et al. 2007; Han et al. 2014). To date, all useful alien genes that significantly contributed to wheat improvement are, without exception, compensating translocations (Friebe et al. 1996; Gill et al. 2006; Han et al. 2014). Thus, understanding the genetic structure of the *A. cristatum* chromosome set may be helpful for producing introgressions or compensating translocations for gene transfer into the wheat genome. In this work, the cDNA FISH probes were distributed unequally along the arms of most chromosomes of *A. cristatum*, leaving some parts uncovered. For instance, the arms 1PL and 6PS have markers only at proximal regions. Thus, more markers are required at certain genomic positions to cover whole chromosome arms of *A. cristatum*. These results provide resources for further studies of the *A. cristatum* genome structure and evolution and contribute to the identification and utilization of wheat-*A. cristatum* derivatives for wheat improvement.

## General discussion, conclusions and future prospects

Prior to this study, several attempts were made to establish a standard karyotype for *A. cristatum.* Generally, the chromosomes of *A. cristatum* were arranged according to their size and centromere position (Asghari et al. 2007; Yang et al. 2014), without considering cross-genome homoeology to Triticeae. In our study, the chromosomes of *A. cristatum* were morphologically characterized and arranged using accurate data on the homoeology between *A. cristatum* and wheat. The molecular karyotype developed in this work enables the identification of an introduced *A. cristatum* chromosome or its segment in the wheat background. Moreover, with the knowledge of genome collinearity with wheat, breeders may predict the probability of induced homoeologous recombination with wheat or other Triticeae. *A. cristatum* was believed to be a species complex of high genetic isolation from other genera of Triticeae (Martin et al. 1999). Nevertheless, our data show a considerable synteny (75.6%) between diploid *A. cristatum* and wheat. Hybridization between wheat and tetraploid *A. cristatum* can be easily done (Chen et al. 1989). To date, all the introgressions from *A. cristatum* into wheat were made using tetraploid *A. cristatum* that originates from derivatives of hybridizations between diploid *Agropyron cristatum* and *Agropyron mongolicum* (Han et al. 2014). Although the diploids *A. cristatum* and *A. mongolicum* contain the same basic P genome, their P genomes exhibited rearrangements and variation (Hsiao et al. 1989; Wu et al. 2006). The two P genomes exhibit segmental autonomy in the tetraploid *A. cristatum* (Stebbins 1947) and are distinguished from each other by structural rearrangements (Hsiao et al. 1989). Schulz-Schaeffer et al. (1963) proposed the segmental alloploid nature of tetraploid and hexaploid *A. cristatum*. Studies of genetic diversity showed that there are genetic variations among different individuals within one population (Liu et al. 2010; Wang et al. 2010); thus, there might be large structural chromosome rearrangements and genetic differences between diploid and tetraploid *A. cristatum*. Further analysis of chromosome structure and rearrangements of tetraploid *A. cristatum,* which is commonly used and successfully hybridizes with wheat, is required. The development of the *A. cristatum* karyotype and comparative cDNA chromosome maps in this study will support alien chromosome introgressions into wheat and may facilitate the use of crested wheatgrass in alien introgression breeding by enabling the transfer of compensating segments or translocations from the targeted alien chromosome into wheat.

### Author contribution statement

The work presented here was carried out in collaboration between all authors. JD, JV and MS conceived and defined the research theme. BF, BG, MS and TD designed the experiments. EH performed the sequencing of DNA, bioinformatics analysis and development of tandem repeat sequences. JČ and TD also worked on laboratory experiments. MK, MS and TD discussed FISH with cDNA methods. EH, JD, MS and TD discussed the results. MS designed the methods, carried out the experiments, analyzed the data and wrote the manuscript. All authors have contributed to, seen, read and approved the manuscript.

## Electronic supplementary material

Below is the link to the electronic supplementary material.

Supplementary material 1 (DOCX 5408 kb)

Supplementary material 2 (DOCX 42 kb)
