# Supplementary material for: The Agropyron cristatum karyotype, chromosome structure and cross-genome homoeology as revealed by fluorescence in situ hybridization with tandem repeats and wheat single-gene probes
Source: Theor Appl Genet. 2018 Aug 1;131(10):2213–27. doi: 10.1007/s00122-018-3148-9 (PMC6154037; doi:10.1007/s00122-018-3148-9)
Supplement: Supplementary file 1 — Supplementary material 1 (DOCX 5408 kb) [file 122_2018_3148_MOESM1_ESM.docx]

**Theoretical and Applied Genetics**

**The *Agropyron cristatum* karyotype*,* chromosome structure and cross-genome homoeology as revealed by fluorescence *in situ* hybridization with tandem repeats and wheat single-gene probes**

**Mahmoud Said^1,2^, Eva Hřibová^1^, Tatiana V. Danilova^3^, Miroslava Karafiátová^1^, Jana Čížková^1^, Bernd Friebe^3^, Jaroslav Doležel^1^, Bikram S. Gill^3^, Jan Vrána^1*^**

^1^Institute of Experimental Botany, Center of the Region Haná for Biotechnological and Agricultural Research, Šlechtitelů 31, CZ-78371 Olomouc, Czech Republic

^2^Field Crops Research Institute, Agricultural Research Centre, 9 Gamma street, Giza, 12619 Cairo, Egypt

^3^Wheat Genetics Resource Center, Kansas State University, 1712 Claflin Road, 4024 Throckmorton PSC, Manhattan, KS 66506, USA

*Jan Vrána

Email: [Vrana@ueb.cas.cz](mailto:Vrana@ueb.cas.cz)

Tel: +420 585 238 720


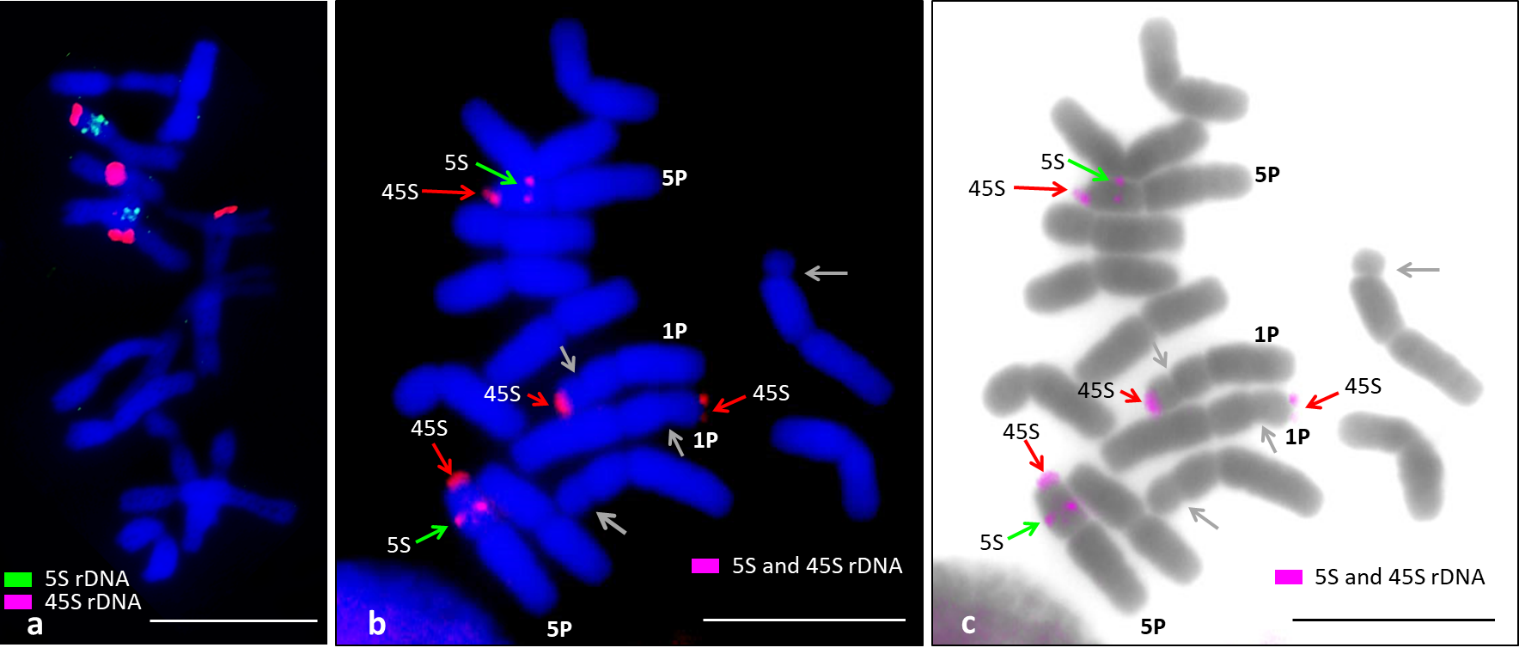


**Supplementary Fig. S1** FISH on mitotic chromosomes of diploid *A. cristatum* cv. Parkway with probes for 5S (green signals “**a**” and green arrows “**b** and **c**”) and 45S (red signals “**a**” and red arrows “**b** and **c**”) rDNA. Two pairs of satellite chromosomes (gray arrows “**b** and **c**”) are shown, one of them lacking 45S rDNA signals. The chromosomes and background are inverted to black and white colors, respectively, for higher definition “**c**”. Bars = 10 μm


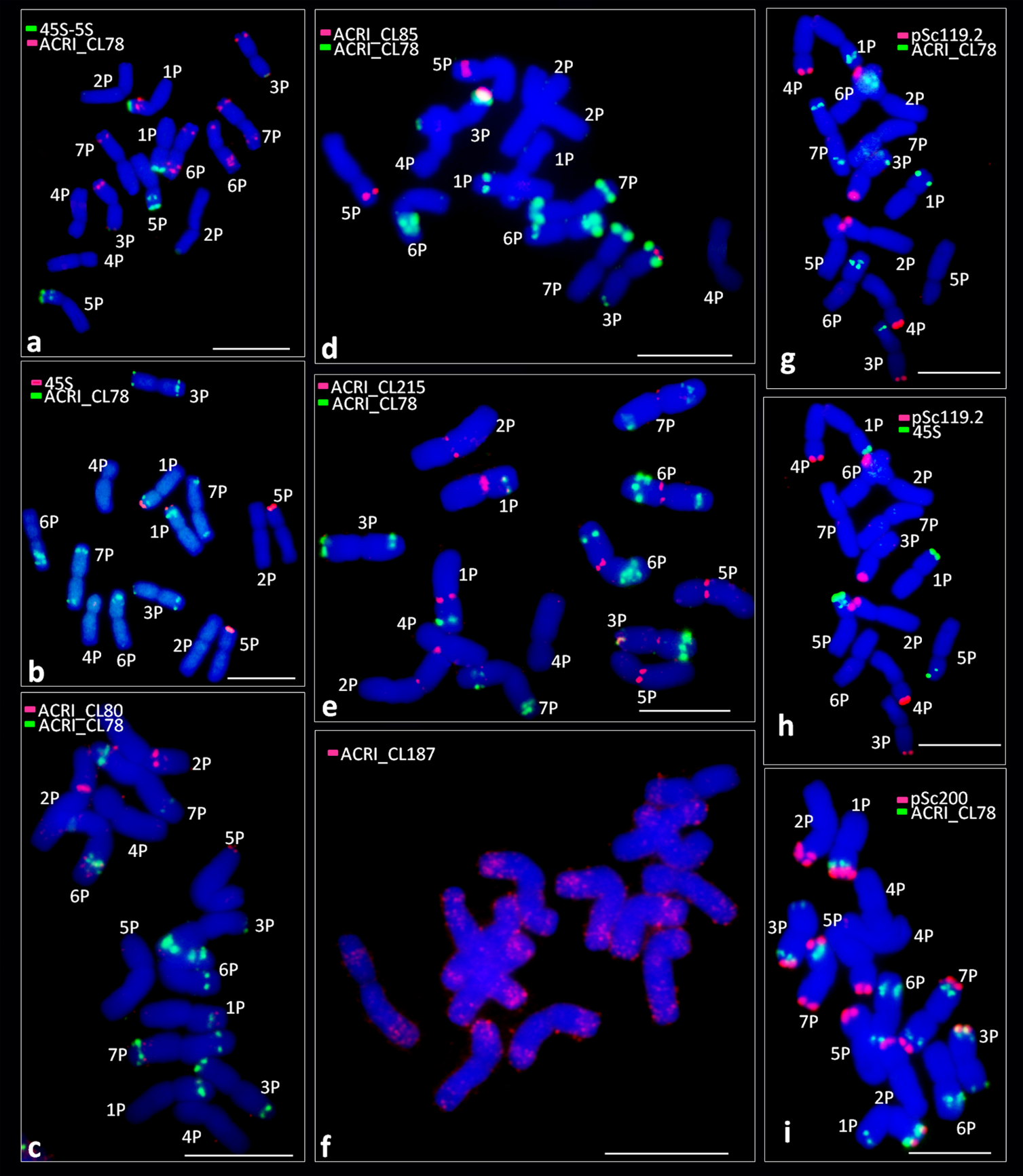


**Supplementary Fig. S2** FISH on mitotic chromosomes of diploid *A. cristatum* cv. Parkway with probes for 5S and 45S rDNA and different tandem repeats. a) 5S and 45S rDNA (green) and ACRI_CL78 (red); b) 45S rDNA (red) and ACRI_CL78 (green); c) ACRI_CL80 (red) and ACRI_CL78 (green); d) ACRI_CL85 (red) and ACRI_CL78 (green); e) ACRI_CL215 (red) and ACRI_CL78 (green); f) ACRI_CL187 (red); g) pSc119.2 (red) and ACRI_CL78 (green); h) pSc119.2 (red) and 45S (green); and i) pSc200 (red) and ACRI_CL78 (green). The chromosomes were counterstained with DAPI (blue). Bars = 10 μm

**
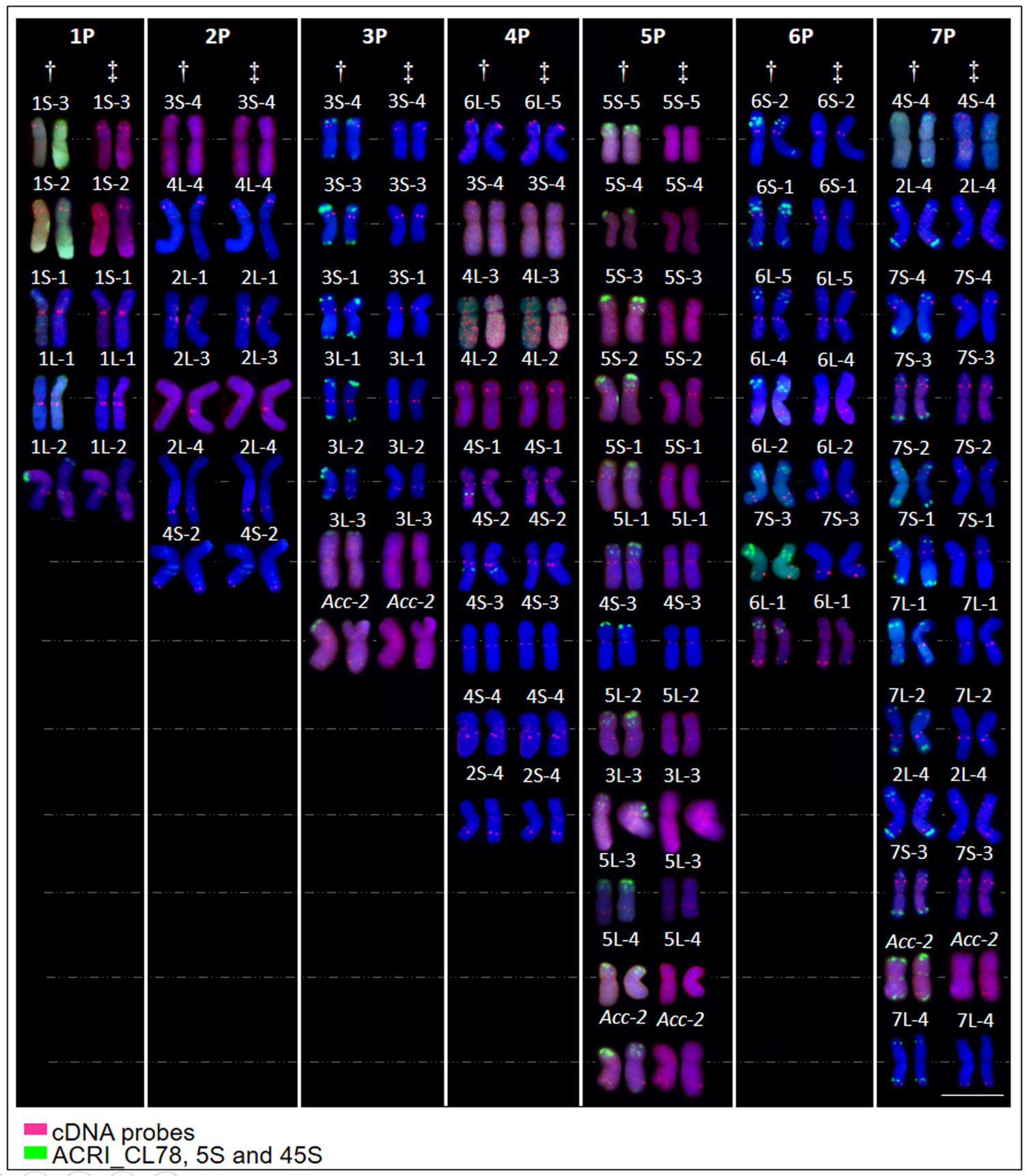
**

**Supplementary Fig. S3** FISH on mitotic chromosomes of diploid *A. cristatum* cv. Parkway shows the distribution of 45 wheat cDNA probes (red dots), in addition to 45S and tandem repeat ACRI_CL78 (both in green). Each chromosome pair (1P-7P) is shown twice; with the patterns of the repeats in addition to cDNA (^†^left of the column) and with only the cDNA probes (^‡^right of the column). The names of the cDNA probes are mentioned above each chromosome pair (Please refer to the online version for the high-resolution figure). Bar = 10 μm

**
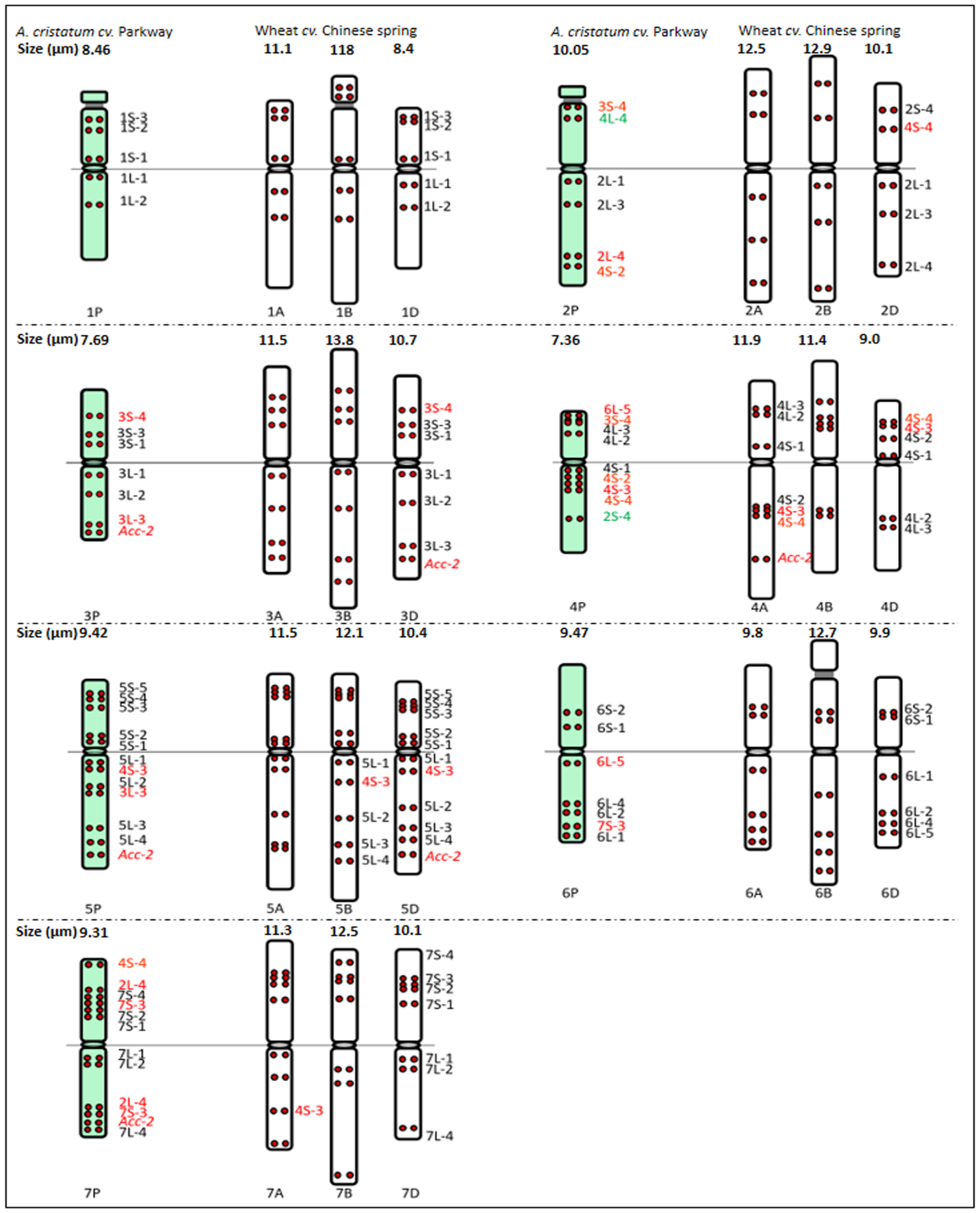
**

**Supplementary Fig. S4** Idiogram showing cDNA positions on diploid *A. cristatum* (cv.) Parkway chromosomes (left), and homoeologous chromosome groups from hexaploid wheat *cv.* Chinese spring (right). The cDNA probe positions are shown as red dots. The names of the probes that hybridized to more than one chromosome are highlighted in red*.* The names of the probes that hybridized to only a non-homoeologous chromosome are highlighted in green. The wheat idiogram and the cDNA positions on wheat chromosomes are from the data published in (Gill *et al*. 1991) and (Danilova *et al*. 2014), respectively
