# Supplementary material for: The Agropyron cristatum karyotype, chromosome structure and cross-genome homoeology as revealed by fluorescence in situ hybridization with tandem repeats and wheat single-gene probes
Source: Theor Appl Genet. 2018 Aug 1;131(10):2213–27. doi: 10.1007/s00122-018-3148-9 (PMC6154037; doi:10.1007/s00122-018-3148-9)
Supplement: Supplementary file 2 — Supplementary material 2 (DOCX 42 kb) [file 122_2018_3148_MOESM2_ESM.docx]

**Theoretical and Applied Genetics**

**The *Agropyron cristatum* karyotype*,* chromosome structure and cross-genome homoeology as revealed by fluorescence *in situ* hybridization with tandem repeats and wheat single-gene probes**

**Mahmoud Said^1,2^, Eva Hřibová^1^, Tatiana V. Danilova^3^, Miroslava Karafiátová^1^, Jana Čížková^1^, Bernd Friebe^3^, Jaroslav Doležel^1^, Bikram S. Gill^3^, Jan Vrána^1*^**

^1^Institute of Experimental Botany, Center of the Region Haná for Biotechnological and Agricultural Research, Šlechtitelů 31, CZ-78371 Olomouc, Czech Republic

^2^Field Crops Research Institute, Agricultural Research Centre, 9 Gamma street, Giza, 12619 Cairo, Egypt

^3^Wheat Genetics Resource Center, Kansas State University, 1712 Claflin Road, 4024 Throckmorton PSC, Manhattan, KS 66506, USA

*Jan Vrána

Email: [Vrana@ueb.cas.cz](mailto:Vrana@ueb.cas.cz)

Tel: +420 585 238 720

**Supplementary Table S1** Primer sequences of the new tandem repeats developed after Illumina sequencing of diploid *A. cristatum* cv. Parkway

| Primer Name | Sequence (5'-3') |
| --- | --- |
| ACRI_CL54_L | CAAGGCAACACAAAATGGAA |
| ACRI_CL54_R | AAAATATCGCACTGCCACCT |
| ACRI_CL76_L | CCTGTGTAGCCCCTTGGTAA |
| ACRI_CL76_R | GAGACCGATTTGAGGGAGTG |
| ACRI_CL78_L | GCGCTTCAATGTACCATTTTG |
| ACRI_CL78_R | CTCGTTGCGATCGTTTTG |
| ACRI_CL80_L | ATGGCCAATGTTATGGCAAC |
| ACRI_CL80_R | AAATGATTTGGTCCAATTTTGC |
| ACRI_CL85_L | AAACGGGCATTTTTCTTTTG |
| ACRI_CL85_R | GCGGAATCGAAGAAATTGTG |
| ACRI_CL86_L | TTTGAATTATGGACAGCACACA |
| ACRI_CL86_R | CCGGCGTAAGCCATATATCA |
| ACRI_CL88_L | AGACGCAACCACCATCCTAC |
| ACRI_CL88_R | CAGAGCGTCATCGACATGAT |
| ACRI_CL128_L | TCTTCACTACCACTGTGATGTTCA |
| ACRI_CL128_R | CCCTGCGTAAGTACATGGAAA |
| ACRI_CL187_L | CGACCGTTAAAATCCTCGAA |
| ACRI_CL187_R | GGAGCAACAGGACCAACACT |
| ACRI_CL198_L | ACGTACCGGACGGTGGAT |
| ACRI_CL198_R | ACGACACGCATCCAGTCAT |
| ACRI_CL215_L | GCGCAAGCTGGGAGTATT |
| ACRI_CL215_R | ATAGCGAGGACGCAAAAA |

**Supplementary Table S2** Chromosome measurements of diploid *A. cristatum* cv. Parkway

| Chromosome | Long arm (L) ± SE  μm | Short arm (S) ± SE  μm | Total length (L+S) = T ± SE  μm | Arm ratio (L/S) | Relative length (T/H) x 100 | Centromeric index (S/T) x100 | Chromosome morphology |
| --- | --- | --- | --- | --- | --- | --- | --- |
| 1P (e) | 4.71 ± 0.07 | 3.75 ± 0.09 | 8.46 ± 0.12 | 1.26 | 13.7 | 44.33 | M^b^ + Sat^c^ |
| 2P (a) | 6.04 ± 0.08 | 4.01 ± 0.08 | 10.05 ± 0.08 | 1.51 | 16.27 | 39.9 | SM^d^ + Sat |
| 3P (f) | 4.07 ± 0.07 | 3.62 ± 0.09 | 7.69 ± 0.12 | 1.12 | 12.45 | 47.07 | M |
| 4P (g) | 4.64 ± 0.08 | 2.72 ± 0.06 | 7.36 ± 0.10 | 1.71 | 11.92 | 36.96 | SM |
| 5P (c) | 5.8 ± 0.10 | 3.62 ± 0.07 | 9.42 ± 0.09 | 1.6 | 15.25 | 38.43 | SM |
| 6P (b) | 4.97 ± 0.11 | 4.5 ± 0.08 | 9.47 ± 0.18 | 1.1 | 15.33 | 47.52 | M |
| 7P (d) | 4.85 ± 0.12 | 4.46 ± 0.10 | 9.31 ± 0.20 | 1.09 | 15.07 | 47.91 | M |
| Total |  |  | 61.76 (H^a^) |  | 100 |  |  |

^a^Total length of the chromosomes in the haploid set

^b^Metacentric chromosome

^c^Satellite chromosome

^d^Sub-metacentric chromosome

**Supplementary Table S3** Localization of full length cDNA probes by FISH on chromosomes of diploid *A. cristatum* cv. Parkway

| FISH probe order on *A. cristatum* | Wheat FISH probe name | Average distance Mean (μm) from the centromere in P genome ± SE | FLcDNA, KOMUGI database | cDNA/probe length, bp |
| --- | --- | --- | --- | --- |
| 1PS-3 | 1S-3 | 2.73 ± 0.06 | tplb0048d21 | 3,487 |
| 1PS-2 | 1S-2 | 2.05 ± 0.02 | AK332649 | 2,860 |
| 1PS-1 | 1S-1 | 0.68 ± 0.08 | AK333586 | 3,522 |
| 1PL-1 | 1L-1 | 0.36 ± 0.03 | tplb0013a02 | 5,094 |
| 1PL-2 | 1L-2 | 1.74 ± 0.04 | tplb0029f23 | 3,113 |
| 2PS-2 | 3S-4 | 3.18 ± 0.03 | tplb0001g16 | 3,127 |
| 2PS-1 | 4L-4 | 2.56 ± 0.01 | AK335609 | 4,790 |
| 2PL-1 | 2L-1 | 0.57 ± 0.03 | tplb0007l09 | 3,165 |
| 2PL-2 | 2L-3 | 1.71 ± 0.01 | tplb0004a16 | 3,841 |
| 2PL-3 | 2L-4 | 4.55 ± 0.07 | AK331687 | 4,036 |
| 2PL-4 | 4S-2 | 4.97 ± 0.02 | tplb0014k23 | 3,488 |
| 3PS-3 | 3S-4 | 2.39 ± 0.03 | tplb0001g16 | 3,127 |
| 3PS-2 | 3S-3 | 1.43 ± 0.01 | tplb0004j16 | 4,402 |
| 3PS-1 | 3S-1 | 1.02 ± 0.03 | tplb0014n06 | 3,237 |
| 3PL-1 | 3L-1 | 0.43 ± 0.03 | AK336104 | 3,860 |
| 3PL-2 | 3L-2 | 1.57 ± 0.03 | tplb0045e08 | 3,369 |
| 3PL-3 | 3L-3 | 3.07 ± 0.01 | AK335612 | 3,596 |
| 3PL-4 | *Acc-2* | 3.43 ± 0.05 |  | 5,592 |
| 4PS-4 | 6L-5 | 2.65 ± 0.08 | tplb0009a09 | 3,283 |
| 4PS-3 | 3S-4 | 2.50 ± 0.07 | tplb0001g16 | 3,127 |
| 4PS-2 | 4L-3 | 2.29 ± 0.06 | tplb0033b21 | 3,024 |
| 4PS-1 | 4L-2 | 1.57 ± 0.08 | AK335837 | 3,866 |
| 4PL-1 | 4S-1 | 0.28 ± 0.03 | AK330261 | 3,582 |
| 4PL-2 | 4S-2 | 0.49 ± 0.06 | tplb0014k23 | 3,488 |
| 4PL-3 | 4S-3 | 0.84 ± 0.03 | tplb0013i03 | 4,240 |
| 4PL-4 | 4S-4 | 1.19 ± 0.04 | tplb0043m19 | 3,384 |
| 4PL-5 | 2S-4 | 2.81 ± 0.01 | tplb0012l12 | 4,143 |
| 5PS-5 | 5S-5 | 3.13 ± 0.01 | tplb0027f03 | 2,416 |
| 5PS-4 | 5S-4 | 2.83 ± 0.07 | tplb0016e11 | 2,847 |
| 5PS-3 | 5S-3 | 2.37 ± 0.01 | tplb0006h03 | 3,807 |
| 5PS-2 | 5S-2 | 0.84 ± 0.06 | tplb0002p18 | 3,112 |
| 5PS-1 | 5S-1 | 0.56 ± 0.03 | tplb0016k09 | 3,057 |
| 5PL-1 | 5L-1 | 0.41 ± 0.03 | tplb0014l23 | 3,737 |
| 5PL-2 | 4S-3 | 0.75 ± 0.03 | tplb0013i03 | 4,240 |
| 5PL-3 | 5L-2 | 1.64 ± 0.04 | AK331808 | 4,808 |
| 5PL-4 | 3L-3 | 1.98 ± 0.01 | AK335612 | 3,596 |
| 5PL-5 | 5L-3 | 3.75 ± 0.04 | AK334748 | 5,408 |
| 5PL-6 | 5L-4 | 4.44 ± 0.02 | tplb0043p15 | 1,514 |
| 5PL-7 | *Acc-2* | 5.05 ± 0.02 |  | 5,592 |

**Supplementary Table S3** continued

| FISH probe on *A. cristatum* | Wheat FISH probe name | Average distance Mean (μm) from the centromere in P genome | FLcDNA, KOMUGI database | cDNA/probe length, bp |
| --- | --- | --- | --- | --- |
| 6PS-2 | 6S-2 | 2.05 ± 0.01 | tplb0006a09 | 3,685 |
| 6PS-1 | 6S-1 | 1.30 ± 0.03 | tplb0050a13 | 3,244 |
| 6PL-1 | 6L-5 | 0.57 ± 0.03 | tplb0009a09 | 3,283 |
| 6PL-2 | 6L-4 | 2.70 ± 0.01 | AK333670 | 4,377 |
| 6PL-3 | 6L-2 | 3.27 ± 0.01 | AK332077 | 5,017 |
| 6PL-4 | 7S-3 | 3.98 ± 0.01 | tplb0006n08 | 3,254 |
| 6PL-5 | 6L-1 | 4.54 ± 0.01 | tplb0016o11 | 2,658 |
| 7PS-6 | 4S-4 | 4.18 ± 0.01 | tplb0043m19 | 3,384 |
| 7PS-5 | 2L-4 | 2.93± 0.03 | AK331687 | 4,036 |
| 7PS-4 | 7S-4 | 2.58 ± 0.01 | tplb0015e09 | 3,640 |
| 7PS-3 | 7S-3 | 2.23 ± 0.01 | tplb0006n08 | 3,254 |
| 7PS-2 | 7S-2 | 1.95 ± 0.01 | tplb0021a05 | 2,889 |
| 7PS-1 | 7S-1 | 1.60 ± 0.03 | AK334430 | 4,404 |
| 7PL-1 | 7L-1 | 0.58 ± 0.03 | tplb0013b07 | 3,360 |
| 7PL-2 | 7L-2 | 0.87 ± 0.03 | tplb0061d08 | 3,147 |
| 7PL-3 | 2L-4 | 3.18 ± 0.01 | AK331687 | 4,036 |
| 7PL-4 | 7S-3 | 3.47 ± 0.02 | tplb0006n08 | 3,254 |
| 7PL-5 | *Acc-2* | 3.98 ± 0.01 |  | 5,592 |
| 7PL-6 | 7L-4 | 4.34 ± 0.01 | tplb0007o14 | 3,957 |

**Supplementary Table S4** Distribution of 45 wheat cDNA probes on chromosomes of *A. cristatum* cv. Parkway

| **cDNA from wheat chromosome Group No.** | cDNA hybridized on homoeologous | cDNA hybridized on homoeologous + non- homoeologous | cDNA hybridized on non-homoeologous | cDNA hybridized on 7P Short + Long arm |
| --- | --- | --- | --- | --- |
| **1** | **5** (S-1, S-2, S-3, L-1, L-2) | **-** | **-** | **-** |
| **2** | **2** (L-1, L-3) | **1** (L-4) | **1** (4L-4) | **-** |
| **3** | **4** (S-1, S-3, L1, L-2) | **3** (S-4, L-3, *Acc2*) |  | **-** |
| **4** | **3** (S-1, L-2, L-3) | **3** (S-2, S-3, S-4) | **1** (2S-4) | **-** |
| **5** | **9** (S-1, S-2, S-3, S-4, S-5, L-1, L-2, L-3, L-4) | **-** | **-** |  |
| **6** | **5** (S-1, S-2, L-1, L-2, L-4) | **1** (L-5) | **-** | **-** |
| **7** | **6** (S-1, S-2, S-4, L-1, L-2, L-4) | **1** (S-3) | **-** | **2** (2L-4, 7S-3) |
| **Total** | **34** (75.6%) amplified on 34 positions | **9** (20%) amplified on 22 positions | **2** (4.4%) amplified on 2 positions | **2** |
